# Cross-cultural validation and reliability of the Turkish version of the anal fistula quality of life scale for cryptoglandular anal fistula

**DOI:** 10.1007/s10151-026-03321-9

**Published:** 2026-05-04

**Authors:** E. Ergüder, S. Leventoğlu, A. Rencüzoğulları, B. Gülcü, B. Menteş, C. Akyol, Ç. E. Şahin, E. Öztürk, H. Pelgur, İ. Aydın, İ. Cem Eray, Ö. Işık, T. Bişgin, U. Özgen, U. Sungurtekin

**Affiliations:** 1 Department of Surgery, Faculty of Medicine, Gazi University, Ankara, Türkiye; 2 Department of Surgery, Ankara Etlik City Hospital, Ankara, Türkiye; 3 Department of Surgery, Koç University Hospital, Istanbul, Türkiye; 4 Department of Surgery, Medicana Bursa Hospital, Bursa, Türkiye; 5 Proctology Unit, Ankara Memorial Hospital, Ankara, Türkiye; 6 Department of Surgery, Ankara University School of Medicine, Ankara, Türkiye; 7 Department of Public Health, Health Sciences University Gulhane Medical Faculty, Ankara, Türkiye; 8 Department of Epidemiology, İstanbul Medipol University Health Sciences Institute, Istanbul, Türkiye; 9 Leventoglu Clinic, Ankara, Türkiye; 10 Department of Surgery, Çukurova University School of Medicine, Adana, Türkiye; 11 Department of Surgery, Uludağ University School of Medicine, Bursa, Türkiye; 12 Department of Surgery, Dokuz Eylül University School of Medicine, İzmir, Türkiye; 13 Department of Surgery, Pamukkale University School of Medicine, Denizli, Türkiye

**Keywords:** Cryptoglandular anal fistula, Quality of life (QoL), Patient-reported outcome measure (PROM), AF-QoL scale, Turkish validation

## Abstract

**Aim:**

The aim of this study was to adapt and validate the Cryptoglandular Anal Fistula Quality of Life (AF-QoL) scale for Turkish-speaking patients, providing a disease-specific, patient-reported outcome measure (PROM) to assess quality of life (QoL) in cryptoglandular anal fistula.

**Methods:**

A prospective, multicenter, cross-sectional study was conducted across seven tertiary centers in Türkiye. The 22-item AF-QoL scale, originally validated in English, was translated and culturally adapted into Turkish following Consensus-Based Standards for the Selection of Health Measurement Instruments (COSMIN) guidelines, including expert consensus and a pilot study (*n* = 10). A total of 220 Turkish-speaking adults with cryptoglandular anal fistula completed the scale. Structural validity was evaluated using exploratory factor analysis (EFA) and confirmatory factor analysis (CFA). Internal consistency was assessed using Cronbach’s alpha, and item–total correlations. Test–retest reliability was evaluated in a clinically stable subgroup of patients.

**Results:**

EFA confirmed a six-domain structure, comprising psychological impact, everyday activities, unpredictability/disease control, seton-related issues, social limitations, and discharge/discomfort, explaining 69.2% of the total variance. CFA demonstrated an acceptable overall model fit (*χ*^2^/*df* = 1.94, root mean square error of approximation [RMSEA] = 0.048, Comparative Fit Index [CFI] = 0.945, standardized root mean square residual [SRMR] = 0.035). The Turkish AF-QoL demonstrated high internal consistency (Cronbach’s *α* = 0.828), satisfactory item–total correlations (*r* = 0.32–0.76), and good test–retest reliability (intraclass correlation coefficient = 0.74), indicating temporal stability.

**Conclusions:**

The Turkish version of the AF-QoL demonstrates satisfactory reliability and structural validity for use in Turkish patients with cryptoglandular anal fistula. This cross-cultural validation provides a disease-specific PROM suitable for clinical and research applications. Further studies assessing additional measurement properties, including structural validity, measurement error, and responsiveness, are warranted.

**Clinical trial registration:**

This study was not a clinical trial and thus was not registered in a clinical trial registry.

**Supplementary Information:**

The online version contains supplementary material available at 10.1007/s10151-026-03321-9.

## What does this paper add to the literature?

**Table:** 

This study provides the first validated Turkish version of the AF-QoL scale for patients with cryptoglandular anal fistula. It fills a gap in disease-specific quality of life measures, and it might support standardized outcome assessment in clinical practice and research in Turkish-speaking populations.	

## Introduction

Anal fistula is a chronic inflammatory condition characterized by an abnormal epithelialized tract connecting the anorectal canal to the perianal skin, typically resulting from cryptoglandular infection [1]. It manifests with symptoms such as pain, purulent discharge, recurrent abscesses, and/or fecal soiling, which severely compromise patients’ quality of life (QoL) owing to chronic discomfort, embarrassment, or social isolation [2, 3]. Given that optimal management of anal fistula aims not only to achieve fistula healing but also enhance patient QoL, assessing QoL outcomes has emerged as an indispensable component in both clinical practice and research [3].

Despite the escalating recognition of QoL as a core outcome measure in anal fistula treatment, there remains a notable deficiency in standardized, disease-specific, patient-reported outcome measures (PROMs) [3]. Generic tools such as the 36-item Short-Form Health Survey (SF-36) and the Fecal Incontinence Quality of Life Scale (FIQL) have been applied in this setting, but they lack the sensitivity to capture the multifaceted QoL impacts unique to cryptoglandular anal fistulas, limiting both reliability and inter-study comparability [4–6]. To address this gap, the Cryptoglandular Anal Fistula Quality of Life (AF-QoL) scale was recently developed and psychometrically validated, demonstrating high reliability (Cronbach’s *α* = 0.927) and validity in English-speaking populations. This patient-centered, 22-item instrument assesses domains including pain, discharge, psychosocial effects, daily activities, and disease unpredictability, reflecting a comprehensive evaluation of fistula-related QoL [3].

Given the absence of a validated, disease-specific QoL tool for cryptoglandular anal fistulas in Türkiye, this study aimed to conduct the linguistic and cultural validation of the AF-QoL scale for Turkish-speaking patients. This adaptation is expected to ensure a robust assessment of QoL impacts, support precise clinical evaluations, advance patient-centered care, and improve the consistency of outcomes in clinical studies and comparative research among Turkish patients with cryptoglandular anal fistulas. Furthermore, by extending the AF-QoL to a new linguistic and cultural context, this study contributes to its international generalizability and facilitates cross-cultural comparisons in colorectal surgery research.

## Methods

### Study design and participants

The current research was a prospective, multicenter, cross-sectional validation and reliability study conducted to adapt and validate the Anal Fistula Quality of Life (AF-QoL) scale for Turkish-speaking patients with cryptoglandular anal fistula. Eligible participants were Turkish-speaking adults aged 18 years or older, diagnosed with cryptoglandular anal fistula, and capable of providing informed consent. Patients with anal fistulas associated with Crohn’s disease and anal fistulas due to anal fissure were excluded to maintain homogeneity with the original study’s target population [3]. Participants were recruited from general surgery and proctology clinics of seven tertiary centers in Türkiye: Gazi University Faculty of Medicine, Ankara Etlik City Hospital, Memorial Ankara Hospital, Bursa Medicana Hospital, Dokuz Eylül University Faculty of Medicine, Çukurova University Faculty of Medicine, and Pamukkale University Faculty of Medicine.

The sample size was calculated on the basis of the 22-item structure of the AF-QoL scale, adhering to the guideline of ten participants per item for robust psychometric analysis [7]. Accordingly, a minimum of 220 participants was required to ensure adequate power for the validity and reliability analyses.

### Translation and cultural adaptation

The AF-QoL scale, originally developed in English by Iqbal et al. [3], was translated into Turkish following a rigorous process to ensure conceptual equivalence and cultural relevance. The translation adhered to the Consensus-Based Standards for the Selection of Health Measurement Instruments (COSMIN) guidelines [8]. First, two independent translators fluent in English and Turkish, with expertise in colorectal surgery and PROM development, translated the scale into Turkish. Medical terms specific to anal fistula (e.g., seton, discharge) and quality-of-life constructs were carefully adapted to align with expressions commonly used in Turkish clinical practice and society.

A consensus meeting involving the translators and a colorectal surgeon was conducted to resolve discrepancies and produce a preliminary Turkish version. This version was back-translated into English by a third expert, blinded to the original scale, to verify its accuracy. The back-translated version was compared with the original English scale to ensure fidelity. Content validity was assessed using the Davis method [9], where a panel of experts (colorectal surgeons and PROM specialists) rated each item as “appropriate,” “requires minor revision,” “requires major revision,” or “inappropriate.” Items achieving ≥ 80% agreement as “appropriate” or “requires minor revision” were retained.

A pilot study was conducted with ten Turkish-speaking patients with cryptoglandular anal fistula from Gazi University Faculty of Medicine, Ankara Etlik City Hospital, and Memorial Ankara Hospital. Participants completed the draft Turkish AF-QoL scale and provided feedback on clarity, comprehensibility, and cultural relevance via semi-structured interviews. Minor revisions to wording were made on the basis of this feedback to enhance readability and acceptability, with no items removed or substantively modified as a result of the pilot phase.

### Study workflow and data collection

The final 22-item Turkish AF-QoL scale was administered to 220 participants across the seven centers. The scale retained the six-factor structure identified in the original study: psychological impact, impact on everyday activities, unpredictability/disease control, seton, impact on going out, and discomfort/discharge [3]. Each item was scored on a five-point Likert scale (0, 25, 50, 75, and 100), with the total score given as a range of 0–100, where higher scores indicate better QoL. For items with a “not applicable” option (e.g., seton-related questions), the overall score was calculated as the average of applicable responses, consistent with the original methodology [3]. Responses marked as “not applicable” were treated as missing data at the item level and were excluded from item-level psychometric analyses. No data imputation methods were applied. Item-level missing data were minimal, and no systematic missingness was observed.

Participants completed the AF-QoL scale, while demographic and clinical data (e.g., age, sex, fistula duration, operative situation) were collected to characterize the study population.

### Statistical analysis

Descriptive statistics were used to summarize the participants’ demographics, reported as the mean ± standard deviation (SD), median with range, and percentage distribution. The psychometric properties of the Turkish AF-QoL scale were evaluated as follows:

1. Validity
  o Content validity: Assessed via the Davis method during the translation phase [9].
  o Structural validity: Evaluated to test the internal structure of the scale. Exploratory factor analysis (EFA) was first conducted to explore the factor structure, followed by confirmatory factor analysis (CFA) to test the fit of the identified model. CFA model fit was assessed using multiple indices (chi-squared [*χ*^2^]/degrees of freedom [*df*], root mean square error of approximation [RMSEA], standardized root mean square residual [SRMR], Comparative Fit Index [CFI], Incremental Fit Index [IFI], and Non-Normed Fit Index [NNFI]/Tucker–Lewis Index [TLI]). In line with contemporary recommendations, model adequacy was primarily judged on the basis of the RMSEA, CFI/TLI, and SRMR, while the Goodness-of-Fit Index (GFI) and Adjusted GFI (AGFI) were reported and interpreted cautiously owing to their sensitivity to sample size and model complexity.
  o Construct validity: using comparator instruments (e.g., convergent/divergent validity) was not assessed in this study and is planned for future research.
2. Reliability
  o Internal consistency: Cronbach’s alpha was computed for the overall scale and each factor, with *α* ≥ 0.70 considered acceptable [10].
  o Item–total correlation (ITC): ITC values ≥ 0.30 were deemed adequate for item retention.
  o Test–retest reliability: A subset of 50 participants completed the scale again after 2–4 weeks, with symptom stability confirmed by patient self-reporting and clinical judgment. This interval was selected to minimize recall bias while assuming clinical stability in patients with chronic cryptoglandular anal fistula, in line with commonly accepted recommendations for PROM reliability testing. The intraclass correlation coefficient (ICC) was calculated and found to be ≥ 0.70, indicating good reliability [10].

Statistical significance was accepted as *p* < 0.05. Analyses were conducted using IBM SPSS Statistics for Windows 26.0 (IBM Corp., Armonk, NY, USA) and Analysis of Moment Structures (AMOS, IBM Corp., Armonk, NY, USA) for CFA.

### Ethical considerations

The study was approved by the Ethics Committee of Ankara Etlik Hospital (AEŞH-BADEK-20241255) and conducted in accordance with the Declaration of Helsinki. Written informed consent was obtained from all participants, and all data were anonymized to ensure confidentiality. Although the study was not preregistered, which is common for cross-sectional validation studies, it was designed and reported in strict adherence to COSMIN guidelines to guarantee transparency and reproducibility.

## Results

A total of 220 Turkish-speaking patients with cryptoglandular anal fistula participated in the validation study. The participants’ demographics and clinical characteristics are summarized in Table 1.

**Table 1:** Demographic and clinical characteristics of the participants

	*N* (%)	Mean (SD)	Min	Max
Age	220	43.3 (11.4)	19	79
Sex				
Male	171 (77.7)			
Female	49 (22.3)			
Operation status				
Pre-op	16 (7.3)			
Post-op	204 (92.7)			

### Factor structure

**Fig. 1:**
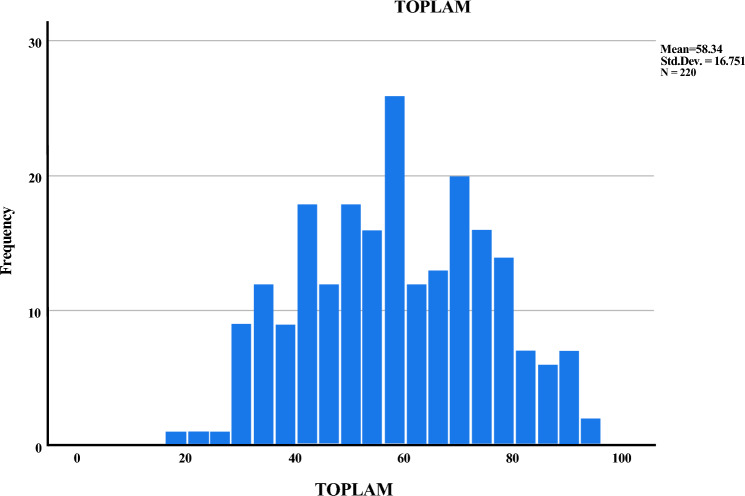
Histogram of the AF-QoL score distribution

EFA was conducted to examine the factor structure of the 22-item Turkish AF-QoL scale (Fig. 1, Table 2). The Kaiser–Meyer–Olkin (KMO) measure was 0.828, exceeding the recommended threshold of 0.70, and Bartlett’s test of sphericity was significant (*χ*^2^(210) = 2107.209, *p* < 0.001), confirming adequate sampling.

**Table 2:** Explained variance by the factors (six subgroups)

	Initial eigenvalues	Degree of variance (%)	Cumulative percentage (%)	Extraction—total	Degree of variance (%)	Cumulative percentage (%)	Rotation—total
1	6.214	29.59	29.59	6.214	29.59	29.59	4.274
2	2.69	12.808	42.398	2.69	12.808	42.398	2.681
3	1.834	8.733	51.131	1.834	8.733	51.131	1.854
4	1.479	7.045	58.176	1.479	7.045	58.176	3.842
5	1.212	5.772	63.948	1.212	5.772	63.948	4.131
6	1.101	5.241	69.189	1.101	5.241	69.189	1.615
7	0.849	4.043	73.233				
8	0.754	3.592	76.825				
9	0.649	3.088	79.913				
10	0.553	2.631	82.544				
11	0.498	2.374	84.918				
12	0.46	2.189	87.107				

The EFA revealed a six-factor structure, consistent with the original scale [3], explaining 69.19% of the total variance. The domains were psychological impact, impact on everyday activities, unpredictability/disease control, seton, impact on going out, and discomfort/discharge. All factor loadings exceeded 0.40, demonstrating adequate item–factor alignment. Detailed item-level descriptive statistics are presented in Table 3, while the explained variance by the factors is presented in Table 2.

**Table 3:** Mean item scores of the AF-QoL scale

Items	Question	Mean (SD)	Median (min–max)
1	How often have you experienced pain or discharge from your fistula?	39.55 (29.43)	25 (0–100)
2	How severe has the pain from your fistula been?	57.73 (30.55)	50 (0–100)
3	How often have you experienced discharge of fluid, pus, or blood from your fistula?	37.50 (29.89)	25 (0–100)
4	How often have you been worried about not being able to control the passage of wind or leaking stool from your back passage or fistula?	56.48 (34.38)	50 (0–100)
5	How often have you been bothered by your fistula symptoms being random and unpredictable?	47.89 (31.24)	50 (0–100)
6	How often have your fistula symptoms interfered with everyday activity, such as walking, sitting, driving, etc.?	58.64 (29.44)	50 (0–100)
7	How many nights have your fistula symptoms made it difficult for you to sleep?	75.61 (30.72)	100 (0–100)
8	Changing dressings or gauze interferes with my planned social or work-related activities	66.82 (32.60)	75 (0–100)
9	My fistula stops me from, or interferes with playing sports or physical activities	57.84 (31.03)	50 (0–100)
10	How many days have you had to take time off from work or school/university because of your fistula?	60.45 (41.57)	75 (0–100)
11	I find it difficult to commute to and from work and other places because of my fistula	58.98 (29.39)	50 (0–100)
12	I am worried about developing an abscess	44.32 (29.77)	50 (0–100)
13	I am worried that I might need a seton forever	46.14 (32.18)	50 (0–100)
14	I find it harder to manage dressings with a seton in	25.91 (36.14)	0 (0–100)
15	My seton is painful	29.55 (36.73)	0 (0–100)
16	How often have you forgotten that you have a seton in?	34.55 (39.98)	0 (0–100)
17	There are members of my family and/or friends from whom I feel isolated because of my fistula and its impact on my life	67.73 (27.21)	75 (0–100)
18	I worry about the impact that my fistula symptoms are having on my partner	67.95 (30.90)	75 (0–100)
19	My fistula has stopped me from having sex or being intimate	68.86 (29.88)	75 (0–100)
20	I feel embarrassed by my fistula	70.34 (30.38)	75 (0–100)
21	I feel sad and/or upset about my fistula, its symptoms, and the impact they are having on my life	48.75 (33.38)	50 (0–100)
22	I feel guilty about the impact my fistula symptoms are having on my life	81.14 (26.21)	100 (0–100)

**Fig. 2:**
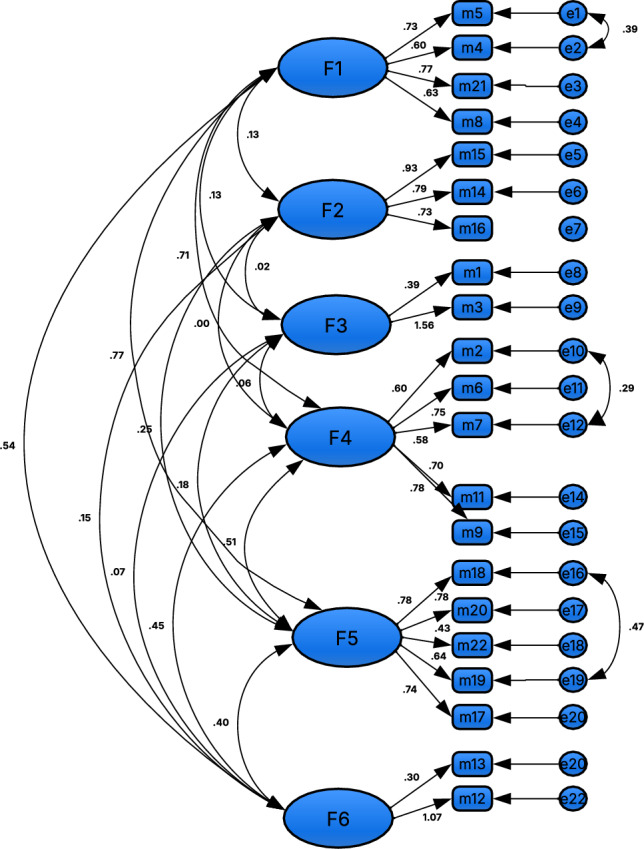
Path diagram of the CFA model

CFA was conducted to evaluate the structural validity of the six-factor model identified in the EFA. The final CFA model demonstrated acceptable fit (*χ*^2^ = 102.6, *df* = 53, *χ*^2^/*df* = 1.94; RMSEA = 0.048; SRMR = 0.035; CFI = 0.945; IFI = 0.946; NNFI/TLI = 0.894). Incremental and residual-based fit indices supported the adequacy of the model. Although absolute fit indices such as GFI (0.766) and AGFI (0.655) were below conventional thresholds, they were interpreted cautiously, and primary focus was placed on the RMSEA, SRMR, and CFI/TLI, which indicated acceptable fit. Therefore, the overall model fit was considered acceptable rather than optimal. Additional fit indices are summarized in Table 4. The CFA path diagram is shown in Fig. 2.

**Table 4:** CFA model fit indices

Fit index	Value
Chi-squared (*χ*^2^)	102.599
*p*-Value	< 0.001
Degrees of freedom (*df*)	53
*χ*^2^/*df*	1.936
RMSEA	0.048
SRMR	0.035
NNFI	0.894
RFI	0.869
CFI	0.945
IFI	0.946
GFI	0.766
AGFI	0.655

*RMSEA*, root mean square error of approximation; *SRMR*, standardized root mean square residual; *NNFI*, Non-Normed Fit Index (also known as the Tucker–Lewis Index), *CFI*, Comparative Fit Index; *IFI*, Incremental Fit Index; *GFI*, Goodness-of-Fit Index; *AGFI*, Adjusted Goodness-of-Fit Index

### Reliability

The Turkish AF-QoL scale demonstrated high internal consistency, with a Cronbach’s alpha of 0.828, exceeding the threshold of 0.70. ITC values ranged from 0.32 to 0.76, confirming that each item contributed meaningfully to the overall structure.

Test–retest reliability was assessed in a subset of 50 patients who completed the scale again within 2–4 weeks. Symptom stability was verified by patient self-report and clinical judgment. The ICC was 0.74, indicating good temporal stability.

These psychometric outcomes closely aligned with the original English validation study (Cronbach’s *α* = 0.927) [3], supporting both the reliability and structural equivalence of the Turkish adaptation.

## Discussion

The AF-QoL scale, originally developed and validated in an English-speaking population by Iqbal et al. [3], has demonstrated excellent psychometric properties (Cronbach’s *α* = 0.927, strong structural validity) as a disease-specific, patient-centered tool for assessing QoL in patients with cryptoglandular anal fistula. However, its applicability to non-English-speaking populations had remained untested until this study. Our adaptation and validation of the AF-QoL scale for Turkish-speaking patients successfully established its reliability and structural validity (Cronbach’s *α* = 0.828), while retaining the original six-factor structure, comprising psychological impact, impact on everyday activities, unpredictability/disease control, seton, impact on going out, and discomfort/discharge. The current study therefore provides Turkish clinicians and researchers with a standardized, culturally relevant PROM to assess QoL, addressing a significant gap in patient-centered outcome measurement for anal fistula in Türkiye.

The Turkish AF-QoL scale fills a critical void in the management of cryptoglandular anal fistula, where symptoms such as pain, discharge, and psychosocial distress profoundly affect patients’ lives. While generic QoL instruments such as SF-36 or FIQL have been used in this context, their lack of sensitivity to fistula-specific impacts has limited their utility [3]. The findings herein confirm that the Turkish adaptation maintains the comprehensive scope of the original instrument, capturing key domains identified through patient input in both studies. As suggested by the authors of the original scale, this study mostly (92.7%) included participants who were in their post-treatment follow-up [3]. Through this study, the instrument’s potential to enhance the accuracy of clinical evaluations, guide treatment decision-making, and contribute to the improvement of patient-centered post-treatment care among Turkish-speaking patients was demonstrated. Importantly, this work directly addresses a major gap in literature, where only 14% of clinical trials have historically assessed QoL outcomes in anal fistula management [11]. Furthermore, the availability of the Turkish AF-QoL will enable future clinical trials in Türkiye to align with international standards for PROM use, thereby improving comparability and the overall quality of fistula-related research.

This study also represents the first application of the AF-QoL scale in a Turkish population, marking an important preliminary step toward its applicability in a different cultural and linguistic context. The retention of the six-factor structure suggests that the core QoL domains affected by cryptoglandular anal fistula are broadly consistent despite cultural differences. This finding suggests that the AF-QoL may capture core QoL domains that are relevant across different cultural settings. In addition, these results provide preliminary support for cross-cultural applicability; however, formal measurement invariance testing is required before firm conclusions regarding generalizability or cross-national comparability can be drawn. Nevertheless, the translation process revealed the need for careful adaptation of terms such as “seton” and “discharge” to reflect Turkish clinical and societal norms, ensuring conceptual equivalence [12]. This cross-cultural validation not only strengthens the AF-QoL’s global relevance but also sets a precedent for further adaptations in other languages, potentially broadening its impact in colorectal surgery research and practice.

Key strengths of this study include its multicenter design across seven Turkish tertiary centers, which enhanced sample diversity, and strict adherence to COSMIN guidelines, mirroring the rigorous methodology of Iqbal et al. [3, 12]. The high internal consistency and acceptable structural validity (e.g., RMSEA = 0.048, CFI = 0.945, SRMR = 0.035) align closely with the original scale, reinforcing the Turkish version’s psychometric integrity. Moreover, the demonstration that AF-QoL retains its structural validity in a non-English-speaking population advances our current understanding by supporting its structural validity in a Turkish-speaking population primarily on the basis of incremental and residual fit indices (RMSEA, CFI, and SRMR), while acknowledging that some absolute fit indices fell below traditional thresholds, and extending its potential applicability to new settings.

This study had several limitations that should be acknowledged. First, while the sample size was adequate for psychometric analysis, all participants were recruited from tertiary centers, which may limit the generalizability of the findings to primary care or rural populations. Unlike the original study (Scale-Content Validity Index, S-CVI/Ave = 0.88), the overall Scale-Content Validity Index was not calculated, although content validity was ensured via the Davis method [9]. In addition, structural validity through hypothesis testing using comparator PROMs (e.g., SF-36, FIQL, or Perineal Disease Activity Index [PDAI]) was not assessed. This was primarily owing to the lack of validated Turkish versions of disease-specific comparator instruments at the time of study design and the intention to minimize respondent burden in a multicenter clinical setting. Future studies should address this limitation by testing predefined hypotheses and evaluating correlations with appropriate generic or disease-specific instruments. Measurement error parameters, such as the standard error of measurement, smallest detectable change, or limits of agreement, were not calculated in this study. Although test–retest reliability was assessed using intraclass correlation coefficients, the absence of measurement error estimates limits the interpretation of individual-level change scores. Therefore, changes observed at the individual patient level should be interpreted with caution until measurement error parameters are established in future studies. Additionally, excluding Crohn’s disease-related fistulas, consistent with Iqbal et al. [3], limits applicability to that subgroup, warranting future investigation. Notably, a separate disease-specific tool, the Crohn’s Anal Fistula Quality of Life (CAF-QoL) scale, has been developed and validated for patients with Crohn’s-associated perianal fistulas, addressing the unique clinical and psychosocial aspects of that condition [13]. Lastly, cultural adaptation required nuanced translation of terms such as “seton” and “discharge,” which may carry different connotations across dialects or regions, potentially affecting interpretation despite pilot testing. Another limitation is the lack of assessment of responsiveness to clinical change, which should be evaluated in future longitudinal studies. Despite these limitations, this study provides a reliable and valid QoL assessment tool for Turkish-speaking patients, offering valuable contributions to both clinical practice and future research.

The implications of this work are substantial. In clinical practice, the Turkish AF-QoL can help identify QoL changes linked to disease progression or treatment, thereby facilitating tailored interventions [3]. Although this study did not evaluate the responsiveness of the Turkish AF-QoL scale, future longitudinal studies may further assess its ability to detect clinical changes over time, particularly in pre- and post-treatment settings. In research, it addresses the historical underrepresentation of QoL in anal fistula studies and supports comparative effectiveness research among Turkish patients. By providing a validated, culturally adapted tool, this study enhances the consistency of QoL outcomes, aligning with the international consensus on the importance of patient-reported outcomes [14].

In conclusion, the Turkish AF-QoL is a reliable and valid PROM that extends the original scale to a new cultural context. It enables robust assessment of QoL in Turkish patients with cryptoglandular anal fistula, supports patient-centered care, and supports future efforts toward international comparability in colorectal research.

## Supplementary Information

Below is the link to the electronic supplementary material.

Supplementary file1 (PDF 605 KB)

## Data Availability

No datasets were generated or analyzed during the current study.

## Declarations

### Permission to reproduce material from other sources

Permission to translate and adapt the original Anal Fistula Quality of Life (AF-QoL) scale was obtained from the corresponding author of the original development study, Dr. Nusrat Iqbal, prior to the commencement of this study.
