# Supplementary material for: Cross-cultural validation and reliability of the Turkish version of the anal fistula quality of life scale for cryptoglandular anal fistula
Source: Tech Coloproctol. 2026 May 4;30(1):81. doi: 10.1007/s10151-026-03321-9 (PMC13284027; doi:10.1007/s10151-026-03321-9)
Supplement: Supplementary file 1 — Supplementary file1 (PDF 605 KB) [file 10151_2026_3321_MOESM1_ESM.pdf]

AF-QoL: Anal Fistül Yaşam Kalitesi Ölçeği

Bu anketin amacı, fistülünüzün günlük yaşamınız üzerindeki etkisini ve bu nedenle yapmak zorunda kaldığınız yaşam tarzı değişikliklerini anlamaktır.

Aşağıdaki sorular, fistülünüz ile alakalı olup, size yaşattığı belirtileri ve bunların son 4-6 hafta boyunca hayatınızı nasıl etkilediğine dair sorular sormaktadır. Lütfen tüm soruları yanıtlayınız. Herhangi bir soruyu nasıl yanıtlayacağınızdan emin değilseniz, size en yakın gelen cevabı vermeye çalışın.

Cevabınızı düşünmek için fazla zaman harcamayın, çünkü ilk düşünceleriniz muhtemelen en doğru olanlarıdır. Bu sorulardan herhangi birini yanıtlamak istemiyorsanız, lütfen boş bırakın veya yanıtlamama nedenlerinizi belirtin.

**Bölüm 1: Belirtileriniz**	
Son 4-6 hafta boyunca, ortalama olarak:	
1. Fistülünüzden ağrı veya akıntı ne sıklıkla yaşadınız?	
a. Hiçbir zaman	100
b. Nadiren	75
c. Haftada birkaç kez	50
d. Her gün	25
e. Günde birkaç kez	0
2. Fistül nedeniyle yaşadığınız ağrının şiddetini değerlendirir misiniz?	
a. Hiç ağrı yok	100
b. Hafif ağrı	75
c. Orta derece ağrı	50
d. Ciddi ağrı	25
e. Tahmin edilebilecek en kötü ağrı	0
3. Fistülünüzden sıvı, iltihap veya kan gelmesini ne sıklıkla yaşadınız?	
a. Hiçbir zaman	100
b. Nadiren	75
c. Haftada birkaç kez	50
d. Her gün	25
e. Günde birkaç kez	0
4. Gaz veya dışkı tutamamaktan ya da fistülünüzden sızıntı olmasından ne sıklıkla endişe duydunuz?	
a. Hiçbir zaman	100
b. Nadiren	75
c. Haftada birkaç kez	50
d. Her gün	25
e. Günde birkaç kez	0

5. Fistül kaynaklı şikayetlerinizin rastgele ve öngörülemez olmasından ne sıklıkla rahatsız oldunuz?<br>a. Hiçbir zaman<br>b. Nadiren<br>c. Haftada birkaç kez<br>d. Her gün<br>e. Günde birkaç kez	 <br>100<br>75<br>50<br>25<br>0
**Bölüm 2: Günlük yaşam ve boş zaman aktiviteleri**	
Son 4-6 hafta boyunca ortalama olarak:	
6. Fistüle bağlı şikayetleriniz yürüme, oturma, araba kullanma gibi günlük faaliyetlerinizi hangi sıklıkla (ne kadar) etkiledi?<br>a. Hiçbir zaman<br>b. Nadiren<br>c. Haftada birkaç kez<br>d. Her gün<br>e. Günde birkaç kez	 <br>100<br>75<br>50<br>25<br>0
7. Fistüle bağlı şikayetleriniz kaç gece uyumanızı zorlaştırdı?<br>a. Hiçbir zaman<br>b. Nadiren<br>c. Haftada birkaç gece<br>d. Her gece	 100<br>67<br>33<br>0
Aşağıdaki ifadelere ne ölçüde katılıyorsunuz ?	
8. Pansuman veya gazlı bezi değiştirmek planlı günlük veya iş ile alakalı faaliyetlerimi etkiliyor.<br>a. Hiçbir zaman<br>b. Nadiren<br>c. Haftada birkaç kez<br>d. Her gün<br>e. Günde birkaç kez	 <br>100<br>75<br>50<br>25<br>0
9. Fistülüm fiziksel aktivite veya spor yapmamı engelliyor ya da zorlaştırıyor<br>a. Hiçbir şekilde<br>b. Pek değil<br>c. Biraz<br>d. Oldukça fazla<br>e. Çok fazla / her zaman	 100<br>75<br>50<br>25<br>0

Bölüm 3: İş ve Okul veya Ünivesite	
Eğer çalışmıyorsanız, sıradaki sorularda lütfen “uygun değil” şıkkını işaretleyin<br><br>Son 4-6 hafta içinde, ortalama:	
10. Fistülünüz nedeniyle ne kadar sık iş veya okuldan izin almak zorunda kaldınız?<br>a. Hiçbir zaman<br>b. Nadiren / Ara sıra bir gün<br>c. Birkaç gün, bir haftaya kadar<br>d. Birkaç hafta<br>e. Son 4-6 hafta içinde işe, okula veya üniversiteye hiç gitmedim<br>f. Uygun değil (Çünkü işe veya okula gitmiyorum)	 100<br>75<br>50<br>25<br>0
Aşağıdaki ifadelere ne ölçüde katılıyorsunuz	
11. Fistülüm nedeniyle işe ve diğer yerlere gidip gelmekte zorlanıyorum<br>a. Hiçbir şekilde<br>b. Pek değil<br>c. Biraz<br>d. Oldukça fazla<br>e. Çok fazla / her zaman	 100<br>75<br>50<br>25<br>0
**Bölüm 4. Ameliyat ve tedavi hakkındaki düşünceleriniz**	
Bazı hastalar tedavilerinin bir parçası olarak ameliyat geçirecektir. Potansiyel olarak ameliyat olma hakkında aşağıdaki ifadelerle ne ölçüde katılıyorsunuz?	
12. Apse gelişmesinden endişe duyuyorum.<br>a. Hiçbir şekilde<br>b. Pek değil<br>c. Biraz<br>d. Oldukça fazla<br>e. Çok fazla / her zaman	 100<br>75<br>50<br>25<br>0
13. Ömür boyu setona ihtiyaç duyacağım diye endişeleniyorum<br>a. Hiçbir şekilde<br>b. Pek değil<br>c. Biraz<br>d. Oldukça fazla<br>e. Çok fazla / her zaman<br>f. Uygun değil – çünkü zaten kalıcı bir setonum var veya artık fistülüm yok	 100<br>75<br>50<br>25<br>0
Lütfen aşağıdaki soruları setonunuz varsa yanıtlayın, aksi takdirde her bir cevap için 'Uygun Değil' seçeneğini işaretleyin:<br>Aşağıdaki ifadelere ne ölçüde katılıyorsunuz?	
14. Seton varken pansuman yapmak daha zor geliyor<br>a. Kesinlikle katılmıyorum<br>b. Biraz katılmıyorum<br>c. Ne katılıyorum ne de katılmıyorum<br>d. Biraz katılıyorum<br>e. Kesinlikle katılıyorum<br>f. Uygun değil	 100<br>75<br>50<br>25<br>0

15. Seton varlığı ağrı verici.<br>a. Kesinlikle katılmıyorum<br>b. Biraz katılmıyorum<br>c. Ne katılıyorum ne de katılmıyorum<br>d. Biraz katılıyorum<br>e. Kesinlikle katılıyorum<br>f. Uygun değil	 100<br>75<br>50<br>25<br>0
Unutulan seton puanı	
16. Son 6 hafta içinde, ortalama olarak, setonunuz olduğunu ne kadar süre unuttunuz?<br>a. Hiçbir zaman<br>b. Nadiren<br>c. Haftada birkaç kez<br>d. Her gün<br>e. Günde birkaç kez<br>f. Uygun değil	 100<br>75<br>50<br>25<br>0
**Bölüm 5. İlişkiler üzerine etkisi**	
Aşağıdaki ifadelere ne ölçüde katılıyorsunuz?	
17. Fistülüm ve bunun etkileri nedeniyle ailemden ve/veya arkadaşlarımdan izole olmuş hissediyorum.<br>a. Hiçbir şekilde<br>b. Pek değil<br>c. Biraz<br>d. Oldukça fazla<br>e. Çok fazla / her zaman	 <br>100<br>75<br>50<br>25<br>0
18. Fistül şikayetlerimin partnerimin üzerindeki etkisi konusunda endişeleniyorum<br>a. Hiçbir şekilde<br>b. Pek değil<br>c. Biraz<br>d. Oldukça fazla<br>e. Çok fazla / her zaman	 100<br>75<br>50<br>25<br>0
19. Fistülüm cinsel ilişkiye girmemi veya yakınlaşmamı engelliyor.<br>a. Hiçbir şekilde<br>b. Pek değil<br>c. Biraz<br>d. Oldukça fazla<br>e. Çok fazla / her zaman	 100<br>75<br>50<br>25<br>0
**Bölüm 6. Psikolojik etkisi**	
20. Fistülüm yüzünden utanıyorum.<br>a. Hiçbir şekilde<br>b. Pek değil<br>c. Biraz<br>d. Oldukça fazla<br>e. Çok fazla / her zaman	 100<br>75<br>50<br>25<br>0

21. Fistülüm, belirtileri ve bunların üzerimdeki etkileri yüzünden üzgün ve/veya mutsuz hissediyorum.<br>a. Hiçbir şekilde<br>b. Pek değil<br>c. Biraz<br>d. Oldukça fazla<br>e. Çok fazla / her zaman	 <br>100<br>75<br>50<br>25<br>0
22. Fistül belirtilerinin hayatıma olan etkilerinden dolayı kendimi suçlu hissediyorum<br>a. Hiçbir şekilde<br>b. Pek değil<br>c. Biraz<br>d. Oldukça fazla<br>e. Çok fazla / her zaman	 100<br>75<br>50<br>25<br>0
A) Toplam:<br>B) "Uygun değil" olarak cevaplanan soru sayısı (en fazla 7):<br><br>AF-YKÖ Puanı = A / (22 - B)	
